# Potential Association Between Anabolic Androgenic Steroid Abuse and Pituitary Apoplexy: A Case Report

**DOI:** 10.3389/fendo.2022.890853

**Published:** 2022-07-22

**Authors:** Agne Andriuskeviciute, Giulia Cossu, Adelina Ameti, Georgios Papadakis, Roy Thomas Daniel, Vincent Dunet, Mahmoud Messerer

**Affiliations:** ^1^ Department of Neurosurgery, University Hospital of Lausanne (CHUV), Lausanne, Switzerland; ^2^ Department of Endocrinology, University Hospital of Lausanne (CHUV), Lausanne, Switzerland; ^3^ Department of Radiology, Neuroradiology division, University Hospital of Lausanne (CHUV), Lausanne, Switzerland

**Keywords:** pituitary apoplexy, pituitary neuroendocrine tumor, testosterone abuse, case report, risk factors, anabolic steroid abuse

## Abstract

**Introduction:**

Pituitary apoplexy (PA) is a rare, and potentially life-threatening condition, caused by hemorrhage or infarction into the pituitary gland with a rapid expansion of the contents of the sella turcica, associated with sudden intense headache, neurological and endocrinological deterioration. The identification of risk factors is crucial for prevention and optimal management. Herein we report a case of PA occurring 1 month after the initiation of anabolic androgenic steroid abuse for bodybuilding.

**Case Report:**

A 40-year-old male patient presents with abrupt onset headache associated with left partial third cranial nerve palsy. The MRI shows a sellar lesion involving left cavernous sinus with a heterogenous anterior aspect of the lesion with hemorrhagic zones in favor of PA. Endocrine work-up shows high testosterone level in patient who was using exogenous testosterone without a medical prescription for a month.

**Conclusion:**

We report a case of PA of a pituitary neuroendocrine tumor occurring shortly after AAS. The association between PA and AAS should be considered as a potential risk.

## Introduction

Pituitary apoplexy (PA) is defined as a hemorrhage or infarction of the pituitary mostly within a pituitary adenoma, associated with intense headache of abrupt onset followed by neurological and endocrinological deterioration with or without altered consciousness, although heterogeneity exists with milder constellations (1, 2). It affects 2% to 12% of patients with pituitary neuroendocrine tumors and the majority of cases occur in patients with non-functioning macroadenomas (1, 3–5). Notably, pituitary apoplexy can be the first presentation of a previously undiagnosed macroadenoma (6). Early recognition and management of PA by monitoring fluid and electrolyte levels, administering glucocorticoids awaiting hormonal work-up results, and correcting pituitary hormone deficiencies is a key to achieve a favorable outcome and complete symptoms resolution (7, 8).

The pathophysiology of PA essentially relies on a mismatch between available blood support and increased needs of tumoral metabolism. It can be triggered by hemodynamic instability or increased intracranial pressure, and the most frequent predisposing factors are a previous surgery (especially cardiac and orthopedic surgery) or angiographic procedure, head trauma, use of Gonadotropin-Releasing Hormone (GnRH) agonists, anticoagulation treatment or pregnancy (1, 9–11). In addition, male sex and tumoral characteristics such as size and type of adenoma are potential risk factors according to another retrospective study (12).

Herein we report a case of pituitary apoplexy following anabolic androgenic steroids (AAS) abuse resulting in abnormally high testosterone levels. For four decades now the AAS abuse has spread into general population for occupational purposes such as bodybuilding and image-enhancing. The extent of this problem is difficult to determine when relying on self-reports of illicit activities (13). It was estimated that the highest rate of androgen abuse is among nonelite sports, followed by athletes, prisoners, drug users and high school students (14). While in professional sports The World Antidoping Agency (WADA) is controlling the doping with the highly sensitive urine detection tests, in general community it might be missed or under controlled in the absence of medical prescriptions or follow-up and leading to supraphysiological doses, habituation or dependency (15).

To our knowledge, this is the first case in literature describing the association between PA and exogenous abuse of testosterone. We will discuss the pathophysiology and the potential impact of a supra-therapeutic dose of testosterone on the cardiovascular system in association with PA.

## Case Description

A 40-year-old man presented to the Emergency Room with abrupt-onset headache evolving for the past 10 days and associated with diminished visual acuity of the left eye, ptosis, and diplopia for 5 days. He was otherwise in good health, and he declared using exogenous androgens without medical prescription in the past 1 month. He started a treatment of anabolic steroids (nandrolone, testosterone enanthate, methandienone and stanozolol) 3 weeks before the occurrence of eye symptoms. Anabolic steroids were used for bodybuilding to increase muscle mass.

**Figure 1:**
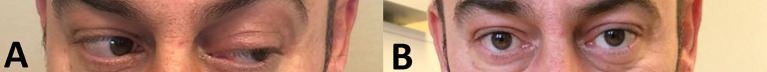
**(A)** Preoperative photo showing left oculomotor palsy with partial ptosis and exaggerated abduction with downward deviation due to unopposed action of trochlear and abducens nerves. **(B)** Post-operative photo showing complete recovery of the left ptosis.

The patient was hemodynamically stable at admission. Clinical examination revealed chemosis of the left eye with partial ptosis. Pupils were isochoric and reactive to light with no deficit in visual acuity or visual fields during the neuro-ophthalmologic initial evaluation. Oculomotor testing revealed deficiency in the left eye adduction with partial paralysis of the oculomotor nerve **Figure 1**. Blood count was normal except for minor leukocytosis without other signs of infection (normal CRP and sedimentation rate). The patient had no clinical signs of cortisol excess or acromegaly. Moreover, he did not exhibit any comorbidities known to be associated with hypercortisolism (diabetes, hypertension, fractures). The hormonal check-up showed excessively high serum testosterone levels (69 nmol/L; normal range: 11-31 nmol/L) associated with markedly reduced LH and FSH levels, consistent with inhibition of gonadal axis by the exogenous androgens. The rest of pituitary work-up was unremarkable **Table 1**. In the absence of a clear consensus in the published guidelines (16), we did not perform routine screening for hypercortisolism in the setting of a pituitary macroadenomas and without any suggestive clinical signs for Cushing syndrome.

**Table 1:** Initial hormonal work-up.

Hormone	At diagnosis	Six weeks post surgery	Normal range
LH	0,4	4,2	2-9 UI/l
FSH	0,6	5,8	2-12 Ul/l
Prolactin	16	11,7	4-16 µg/L
ACTH	25		25 ng/L
TSH	0,8	2,21	0,270 – 4,20 mU/l
T4	12,1	15,8	12 – 22 pmol/L
Testosterone	68,9	13,7	11 – 31 nmol/L
Cortisol			
Basal After cosyntropin stimulation	563	148 768	133 – 537 nmol/L > 500 nmol/l
IGF-1	161		85 – 184 µg/L

LH, luteizing hormone; FSH, Follicle Stimulating Hormone; ACTH, Adrenocorticotropic Hormone; TSH, Thyroid Stimulating Hormone; T4, Thyroxine; IGF-1, Insulin-like growth factor-1.

**Figure 2:**
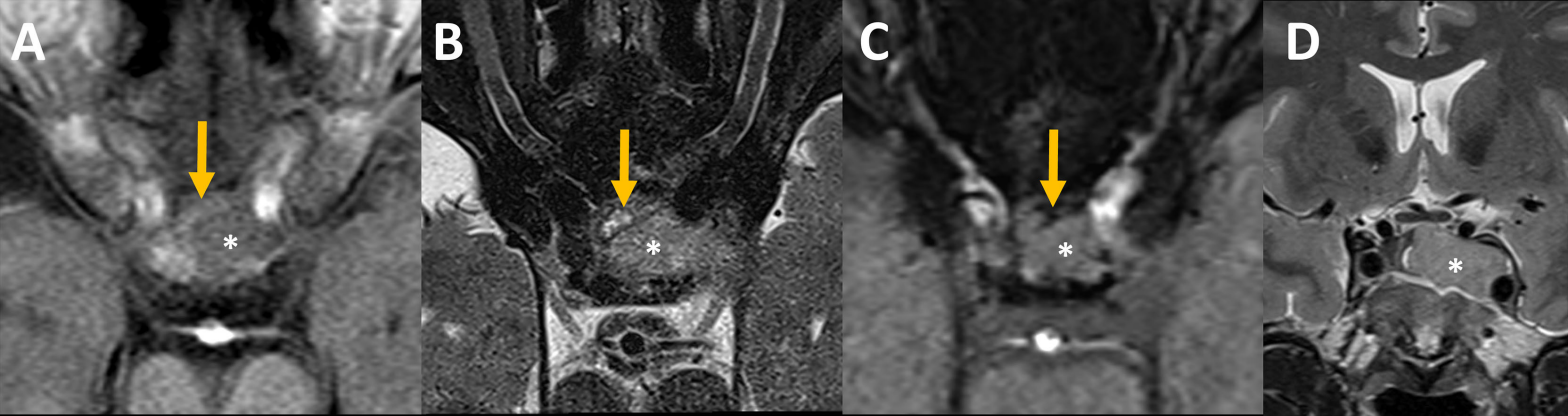
Pre-operative MRI Axial MR imaging focused on the sella at admission **(A–C)**: The intra-sellar lesion (star) was heterogeneous on its anterior aspect. Some bloody components (orange arrows) that appeared slightly hyperintense on unenhanced T1 **(A)**, hyperintense with an hypointense rim on T2 spin echo **(B)** and hypointense on susceptibility weighted images, SWI, **(C)** were intercalated between the bone and the rest of the lesion. **(D)** Coronal MR imaging showed pituitary adenoma with invasion of the left cavernous sinus, grade 3b according to Knosp classification (17).

The initial MRI showed a sellar lesion with an invasion of the left cavernous sinus classified 3b according to Knosp classification (17). A heterogenous anterior aspect of the lesion compatible with hemorrhagic zones was in favor of PA and normal pituitary gland was displaced on the right side **Figure 2**.

After initial conservative management, the further treatment plan was discussed with our multidisciplinary team which includes a referent surgeon for pituitary surgery and neuro-endocrinologists, the patient and his wife. While the choice of surgical treatment versus conservative management remains controversial in the literature when only oculomotor symptoms are present, some data indicate possibly higher rate of ocular palsy recovery after surgical intervention compared to conservative management (18). Taking into consideration that our patient is a young individual engaged in active social and professional life, without any significant comorbidities and with a handicapping ocular palsy, a surgical treatment was decided.

**Figure 3:**
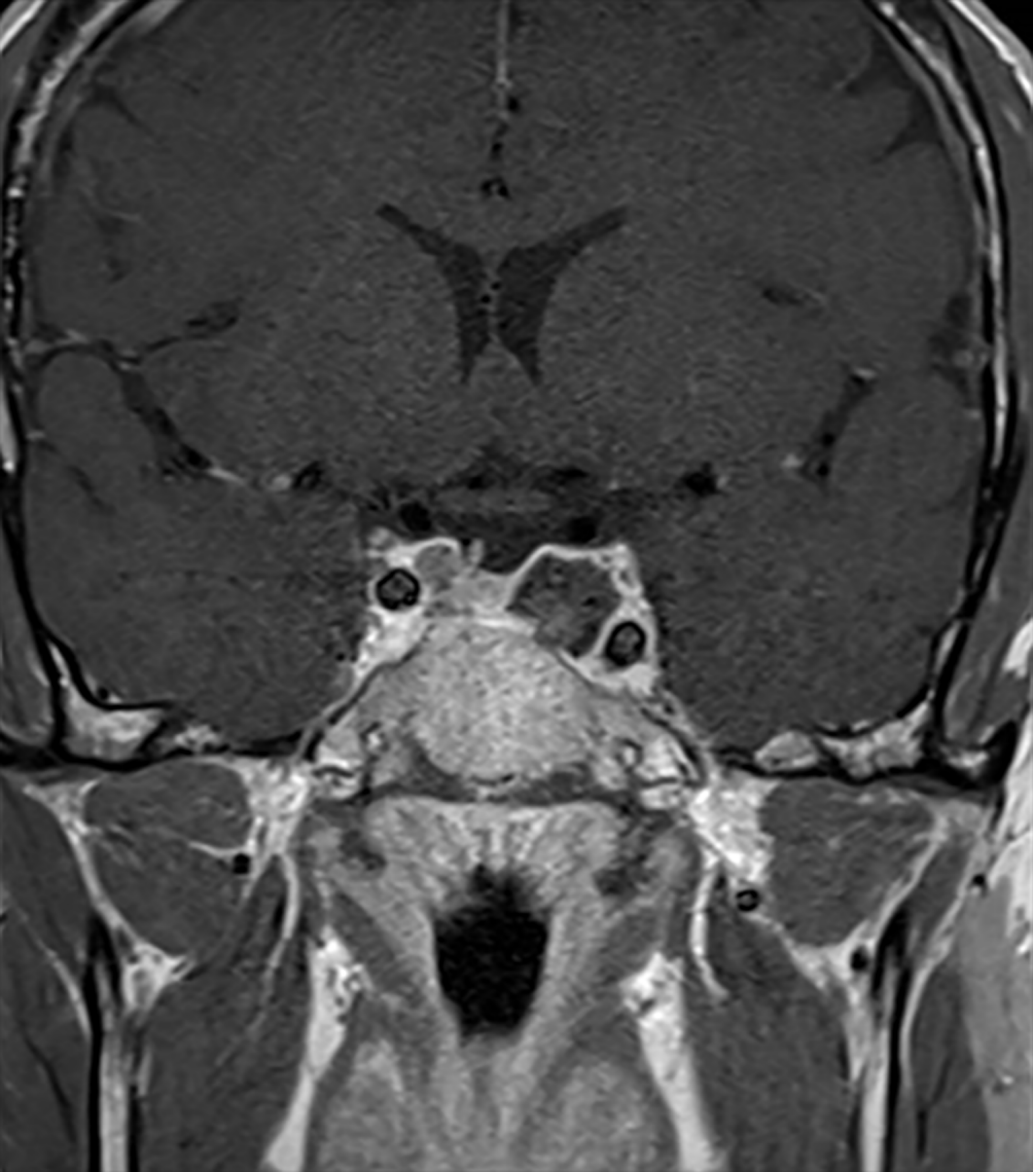
Post-operative MRI. Sagittal MR Imaging T1 with Gadolinium showed a total resection of the pituitary adenoma.

The patient was then operated using an endoscopic endonasal transsphenoidal approach to remove the tumor and decompress the structures inside the cavernous sinus (19). Intraoperatively, we found hemorrhagic areas and blood clots inside the tumor which confirmed the diagnosis of apoplexy. Histopathological analysis revealed the presence of a clinically silent sparsely granulated corticotroph adenoma. There was no hormonal co-expression detected. Post-operatively, third cranial nerve palsy resolved **Figure 1**. His post-operative work-up showed mild/moderate corticotrope deficiency and the patient was temporarily substituted with hydrocortisone. Subsequent pituitary retesting at 6 weeks post surgery confirmed normalized corticotrope function (ACTH-stimulation testing) and reversal of the hypogonadotropic profile in the presence of normal testosterone levels **Table 1**. Immediate postoperative MRI showed no residual tumor **Figure 3**. Given the absence of residual tumor, we did not perform a screening for subclinical hypercortisolism (such as a low-dose dexamethasone suppression test) after the surgery. Serial monitoring of cortisol and ACTH levels is scheduled.

## Discussion

This case describes a potential relationship between AAS abuse and PA which has not been reported before. Drug-induced PA is rare but has been reported with dopamine agonists (such as cabergoline and bromocriptine), anticoagulants, estrogen-based hormonal contraception and GnRH agonists used for prostate cancer (1). The PA is thought to be caused by dynamic hormonal changes resulting in higher metabolic demands with inadequate vascular support due to reduced and abnormal angiogenesis in macroadenomas. For instance, GnRH agonists are associated with PA occurring approximately ten days after the injection in most cases. This is thought to coincide with the initial effect of these medications (flare effect), consisting of an initial elevation in LH, FSH and testosterone before subsequent suppression because of downregulation and desensibilization of GnRH receptors. This initially stimulatory effect could generate an acute growth of metabolic demands especially in non-functioning gonadotropinomas favoring the occurrence of PA (12, 20, 21).

There is conflicting evidence about the impact of testosterone on the cardiovascular system (22). Indeed, testosterone might act as an atheroprotector inducing endothelium-dependent and independent vasodilation and reduction of arterial stiffness. Yet, its administration in animal models has also been shown to lead to vasoconstriction *via* activation of thromboxane A2, renin-angiotensin-aldosterone system, and norepinephrine synthesis (23). Testosterone replacement therapy (TRT) was associated with an increased risk of ischemic stroke, transient ischemic attack, and myocardial infarction (HR 1.21 for composite outcome; 95% CI, 1.00-1.46) in a recent large cohort study (24). Nevertheless, data from retrospective and randomized controlled studies remain conflicting in part due to differences in inclusion criteria (presence or not of hypogonadism at baseline) as well as in the treatment modalities (dose, route of administration) (22). The risk of adverse vascular events associated with exogenous testosterone may be explained by an enhancement of platelet aggregation, thus favoring coronary plaque or thrombus development (25).

While the main indication of TRT is testosterone deficiency with clinical signs of hypogonadism (26), exogenous testosterone, alone or combined with other anabolic steroids, is commonly taken off-label to increase muscle mass, strength, and power (24, 27). The cardiovascular and cerebrovascular toxicity of this practice is well established (28). Indeed, AAS have been shown to confer an enhanced pro-thrombotic state, alter vascular reactivity (including induction of vasospasm) and cause hypertension (29). All the aforementioned mechanisms have been linked to the pathophysiology of PA (1). Furthermore, as in our case, intramuscular injections of testosterone, even when used at a therapeutic setting, cause spikes of plasma testosterone level, which are related to highest rate of complications, especially in the first months of the treatment (24, 30, 31).

The relationship between PA and exogenous use of androgens at supratherapeutic doses has never been reported before. The temporal association between the AAS exposure and the occurrence of apoplexy in our patient, who did not exhibit any other known precipitating factors, suggest that the documented excess of testosterone may have contributed to pituitary ischemia through the cascade of aforementioned mechanisms. In this setting of an individual case report, we cannot prove the causal effect of AAS abuse or exclude a potential role of other factors such as the histologic type of the tumor (32). A certain degree of subclinical hypercortisolism before surgery, contributing to a prothrombotic state, cannot be formally excluded, but seems very unlikely based on recent data from relatively large series of clinically silent corticotroph macroadenomas (33). Given that AAS for non-medical purposes are most often bought over the counter, we cannot exclude the possibility that the AAS preparation in this case could have contained other stimulant substances exerting an additive effect to the prothrombotic state and contributing to the PA. Nevertheless, our observation highlights the possible association between illicit use of testosterone and other androgens and PA.

## Conclusion

We report a case of PA of a pituitary neuroendocrine tumor occurring shortly after AAS abuse that led to supratherapeutic testosterone blood levels. The risk of ischemic complications due to exogenous androgens for non-medical conditions should not be underestimated and PA could be part of the morbidity spectrum. Physicians should conduct a thorough drug history and inquire patients regarding eventual self-medication.

## Data Availability Statement

The original contributions presented in the study are included in the article/**Supplementary Material**. Further inquiries can be directed to the corresponding author.

## Ethics Statement

Written informed consent was obtained from the individual(s) for the publication of any potentially identifiable images or data included in this article.

## Author Contributions

AA, GC wrote the manuscript with support from AdA, GP, VD and RD. All authors provided critical feedback and helped shape the manuscript. All authors contributed to the article and approved the submitted version.

## Funding

This research did not receive any specific grant from any funding agency in the public, commercial or not-for-profit sector. Open access funding was provided by the University of Lausanne.

## Conflict of Interest

The authors declare that the research was conducted in the absence of any commercial or financial relationships that could be construed as a potential conflict of interest.

## Publisher’s Note

All claims expressed in this article are solely those of the authors and do not necessarily represent those of their affiliated organizations, or those of the publisher, the editors and the reviewers. Any product that may be evaluated in this article, or claim that may be made by its manufacturer, is not guaranteed or endorsed by the publisher.
