# Supplementary figures and images for: Potential Association Between Anabolic Androgenic Steroid Abuse and Pituitary Apoplexy: A Case Report
Source: Front Endocrinol (Lausanne). 2022 Jul 22;13:890853. doi: 10.3389/fendo.2022.890853 (PMC9354695; doi:10.3389/fendo.2022.890853)

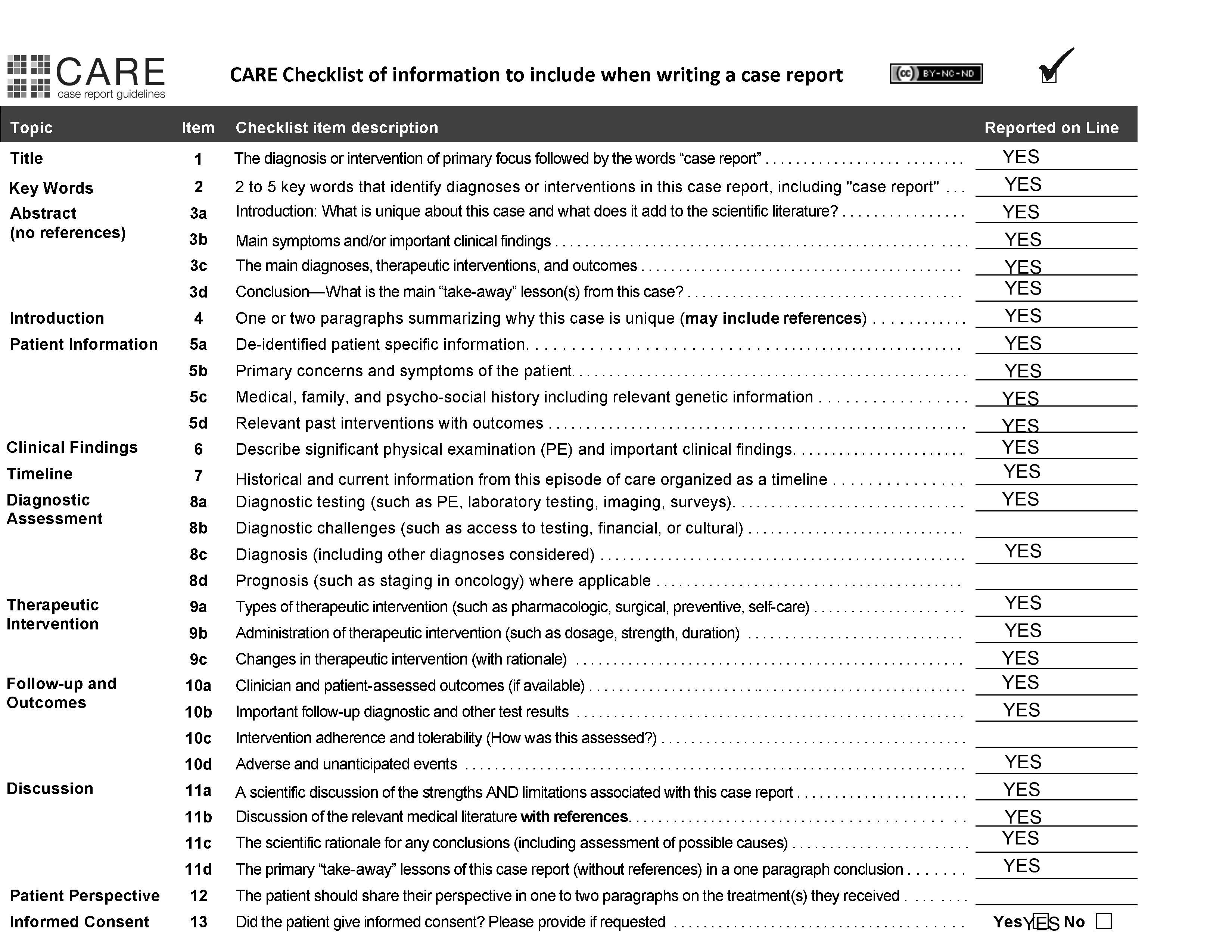

Supplement: Supplementary file 1 [file Image_1.tif]
